# Why addressing conflicts of interest is essential to progress in reducing commercially driven health harms: Lessons from tobacco

**DOI:** 10.1016/j.fhj.2025.100268

**Published:** 2025-06-30

**Authors:** Anna B Gilmore, Rachel A Barry, Alice Fabbri

**Affiliations:** a Centre for 21st Century Public Health, Department for Health, University of Bath, Bath, UK; b UK Population Health Improvement UK (PHI UK) – Local Health and Global Profits, UK

**Keywords:** Conflicts of interest (COIS), Tobacco control, Commercial determinants of health, Lesson learning

## Abstract

Globally and in the UK, significant progress has been made in tackling the harms of tobacco use. This was enabled by addressing the fundamental conflict between the tobacco industry’s interests and those of the public, including by rejecting partnerships with the tobacco industry. Conflicts of interest (COIs), like those identified in tobacco control, exist in other areas of public health, yet governments, including the UK, continue to work in partnership with other health-harming industries, including alcohol, ultra-processed food and gambling, despite evidence that partnership approaches where COIs exist are ineffective. This article details lessons that can be drawn from this experience, outlining how understanding and addressing COIs in policy, professional practice and science are prerequisites to tackling commercially driven harms.

## Introduction

Globally and in the UK, the huge progress made in addressing the harms from tobacco use was enabled by addressing the fundamental conflict between the tobacco industry’s interests and the public interest.^1^ This article details lessons that can be drawn from this experience, outlining how addressing conflicts of interest (COIs) is a prerequisite to tackling commercially driven harms. With just four commercial products – tobacco, fossil fuels, ultra-processed food and alcohol – estimated to cause between a third and almost two third of global deaths, the need to address such harms could not be more urgent.^2^

## History of UK tobacco control

In the UK, efforts to reduce tobacco’s harms began following publication of the first Royal College of Physicians report, *Smoking and health*, in 1962.^3^ Although some advances were made, it took at least 30 years for successive governments to recognise the fundamental conflict between public health goals and the tobacco industry’s financial interests. Instead, they worked in partnership with the tobacco industry and initially took advice from a scientific liaison committee which included industry-nominated scientists,^3^ while a later committee was required to consult with and depended on research from the tobacco industry.^4^ Rather than regulating, which we now know is essential to addressing commercially driven health harms, they relied largely on ineffective voluntary agreements, which the industry regularly breached.^5^

Progress was inevitably limited. For example, the UK government’s first voluntary agreement with the tobacco industry in 1972 included provisions that cigarette packs carry voluntary ‘health hints’ like ‘leave a long stub’.^6^ Although such industry-favourable messaging seems unfathomable now, it was unfettered industry activities like this that led to millions dying unnecessarily.

**Fig. 1:**
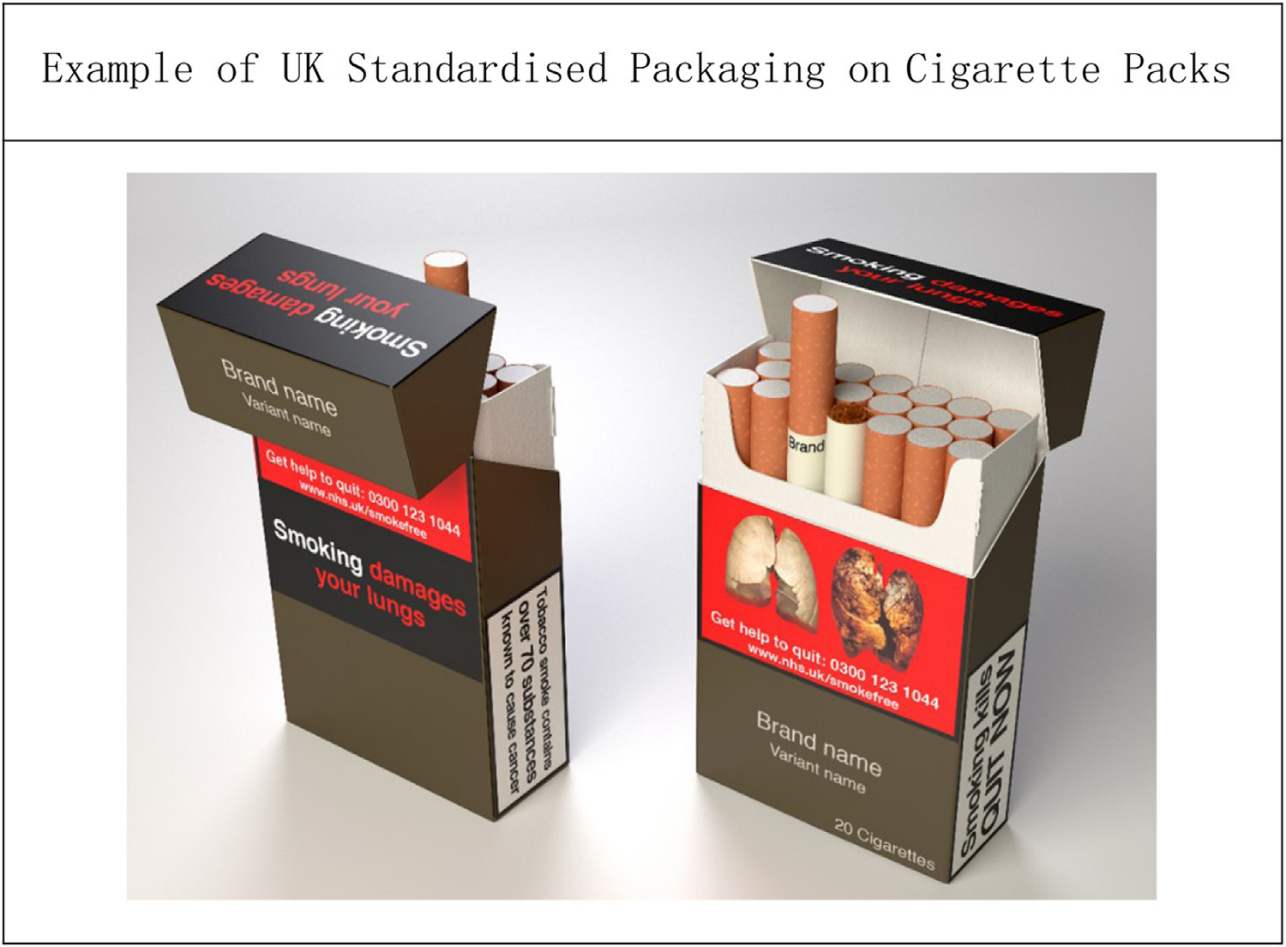
Example of UK standardised packaging on cigarette packs. This material is reproduced from www.TobaccoTactics.org. Copyright © the University of Bath, licensed under CC BY-NC 4.0.

It was not until 1991 that health warning labels were finally made mandatory in the UK, albeit covering just 6% of the pack^3^^,^^4^ – a far cry from the standardised cigarette packaging of today (Fig. 1). Yet even this regulation was prompted by a 1990 European Union directive which Thatcher’s government had opposed.^3^

From that point, implementation of mandatory (enforceable) regulation accelerated. Numerous issues underpinned this change,^7^ among them increased recognition of both the tobacco industry’s role as the vector of the tobacco epidemic and the fundamental conflict between its interests and those of the public.^8^

## What are conflicts of interest?

Broadly, COIs can be both individual and institutional. Individual COIs are ‘*circumstances that create a risk that professional judgements or actions regarding a primary interest will be unduly influenced by a secondary* interest’*.*^9^ Institutional COIs occur instead when there is potential for an organisation’s primary aims to be unduly influenced by the conflicting interest of another body.^10^ In his book *The perils of artnerships*, Marks explains that while public institutions are guardians of the public good, corporations’ primary motivation is to maximise profits.^11^ Collaborations between these two actors therefore create COIs, which can undermine the integrity of a public institution and its ability to deliver on its remit. COIs can therefore exist regardless of the product that a commercial actor produces; an issue which has hitherto not been well understood (Box 1).

Box 1 **Misconceptions about CsOI**

Many have mistakenly understood the conflict of interest that has been recognised and addressed within tobacco control as relating specifically to tobacco and therefore not applicable to other corporations. But tobacco is simply a product, albeit a uniquely harmful one; it does not have ‘interests’, it does not seek to influence policy or science, or to maximise profits. The conflict instead lies between the interests of the tobacco companies (to maximise sales and profits) and the public interest and by extension, therefore, the government’s interest, given its duty to act in the public interest. The product simply determines the *scale of the harm* that arises from failure to address that conflict.

Alt-text: Unlabelled box

## Tobacco control’s paradigm shift from partnership approaches to recognising and addressing conflicts of interest

### Groundbreaking research using internal tobacco industry documents

The paradigm shift towards understanding and addressing COIs in tobacco control came with publication of the first industry document research in 1995.^12^ This laid bare what the tobacco industry was really up to.

A 1989 strategic planning document created for a major UK-based tobacco company showed that tobacco companies knew that voluntary agreements, such as the pack labelling detailed above or its voluntary codes on marketing, were ineffective.^1^ Instead they promoted them not only to preclude effective, mandatory regulation^13^^,^^14^ but to boost their credibility with and access to governments. Tobacco companies then quietly circumvented these toothless agreements.^5^ For example, widespread exposure to tobacco marketing among children was documented after adoption of a voluntary agreement to restrict such marketing in the UK.^15^

Similarly, internal industry documents revealed that the real purpose of the tobacco industry’s so-called ‘youth smoking prevention’ programmes, typically run in partnership with governments, was to fight regulation.^16^ Unsurprisingly, these programmes were found to have either no impact or to increase youth smoking.^17^

More shocking still were the revelations that the industry had known about the carcinogenic and addictive nature of its products for decades. Yet they obfuscated and denied those findings, while simultaneously engineering cigarettes to maximise their addictive potential.^12^^,^^18^ This scientific deception involved both covert science (controlled by lawyers so that findings could be hidden)^19^ and highly publicised ‘distracting’ science intended to *distract* from the harms of tobacco by, for example, focusing on other causes of cancer and heart disease.^12^^,^^18^ Funding leading academics to do this work gave industry the added advantage of securing reputational gains and using these scientists to achieve influence.^20^

### The policy response: The WHO framework convention on tobacco control and Article 5.3

On the policy front, revelations from these documents prompted an important process of tobacco industry denormalisation and helped initiate negotiations, beginning in 1996, for the world’s first public health treaty developed under the World Health Organization (WHO) – the WHO Framework Convention on Tobacco Control.^8^ More specifically, they led to the treaty’s inclusion of Article 5.3, which recognises *‘the fundamental and irreconcilable conflict between the tobacco industry’s interests and public health policy interests’.*^21^ Like the rest of the treaty, this is legally binding, requiring all government departments to protect their public health policies from the tobacco industry, primarily by excluding them from the policymaking process (see Box 2).^21^

Article 5.3 has proved an essential foundation to progress and to reducing industry power and influence.^8^^,^^22^ The UK, for example, now has some of the most advanced tobacco control policies in the world and among the highest levels of Article 5.3 implementation.^23^^,^^24^ Implementation has, nevertheless, been challenging^25^: the tobacco industry increasingly operates through third parties and front groups^24^^,^^26^ and targets individual politicians and civil servants in non-health ministries who have limited knowledge of Article 5.3, amid conflicting mandates.^27^

Box 2 **The four guiding principles and eight primary recommendations of the WHO FCTC Article 5.3 guidelines which detail how governments should implement Article 5.3 (20)**

**The guiding principles:**

• Principle 1: There is a fundamental and irreconcilable conflict between the tobacco industry’s interests and public health policy interests.
• Principle 2: Parties, when dealing with the tobacco industry or those working to further its interests, should be accountable and transparent.
• Principle 3: Parties should require the tobacco industry and those working to further its interests to operate and act in a manner that is accountable and transparent.
• Principle 4: Because their products are lethal, the tobacco industry should not be granted incentives to establish or run their businesses.

**The recommendations:**

1. Raise awareness about the addictive and harmful nature of tobacco products and about tobacco industry interference with Parties’ tobacco control policies.
2. Establish measures to limit interactions with the tobacco industry and ensure the transparency of those interactions that occur.
3. Reject partnerships and non-binding or non-enforceable agreements with the tobacco industry.
4. Avoid conflicts of interest for government officials and employees.
5. Require that information provided by the tobacco industry be transparent and accurate.
6. Denormalise and, to the extent possible, regulate activities described as ‘socially responsible’ by the tobacco industry, including but not limited to activities described as ‘corporate social responsibility’.
7. Do not give preferential treatment to the tobacco industry.
8. Treat state-owned tobacco industry in the same way as any other tobacco industry.

Alt-text: Unlabelled box

### The scientific response: Managing COIs in science

Efforts were also made to counter industry influence on science by either minimising or exposing COIs.^28^ Led largely by the scientific community, not governments, these included some universities refusing to accept research funding from tobacco companies, funders refusing to fund those who also take tobacco industry monies, professional societies precluding industry participation in conferences and journals adopting policies prohibiting the publication of tobacco industry-funded science.^28^

Most journals, however, continue to rely on a combination of peer review and reporting of COIs. While important, the evidence is now clear that such measures are insufficient and easily circumvented.^29^ The best peer review cannot address the distracting research, which can be technically sound, nor can it identify, for example, the industry’s deliberate use of datasets that underestimate the link between second-hand smoke and lung cancer.^30^ Similarly, disclosure of potential COIs does not eliminate those conflicts or reduce the bias that emerges from them, and may even have the opposite effect.^31^ Moreover, corporations circumvent such rules by channelling research funding through third parties while industry-funded authors frequently fail to disclose their COIs.^29^ Consequently, journal editors report practical difficulties in assessing COI declarations.^32^

## Learning from tobacco to address other commercially driven health harms

### Learning from the past: The same COIs

The same COIs that have been addressed over decades in tobacco exist in other areas of public health involving corporate interests, including food and obesity, alcohol, gambling, climate and the environment, and pharmaceuticals. This is because corporations share a common primary remit to maximise profit (see Box 2).^2^^,^^11^ Consistent with this, there is growing evidence – some even based on internal documents of other industries^6^, ^33^, ^34^ – that these other industries engage in the same scientific^20^ and political practices as tobacco,^35^ and for the same purpose – to prevent regulation and litigation, and maximise their product use and profit. Many even worked with tobacco companies to shape the rules on how policies are made, specifically to make it harder to pass public health legislation.^2^ There is also now far greater evidence that voluntary or partnership approaches, including what are now known as multi-stakeholder initiatives which involve corporations in formulating public health policies, do not work where there are conflicts between their interests and the public health goals being pursued.^36^^,^^37^

Unfortunately, successive governments have failed to recognise this mounting evidence and, to the detriment of the UK’s health, continue to work in partnership with these industries.^38^

### Learning from the present: The tobacco industry’s claimed reinvention

Meanwhile, in the tobacco policy space, history is repeating itself because tobacco companies are using their new products (e-cigarettes and nicotine products) to claim that, like these other companies, they deserve a seat at the policy table.^39^ The outcome is that, both globally and in the UK, tobacco industry interference has increased^23^^,^^24^ and progress in tobacco control has stalled.^39^^,^^40^ In the UK not only is youth vaping increasing rapidly, but data suggest that youth smoking is now rising,^41^ as is adult smoking in parts of the country.^42^

### Science ceases to operate in the public interest

In relation to science, recent research shows that the tobacco industry is re-engaging in the very same problematic scientific practices of the past^43^^,^^44^ and the measures detailed above are inadequate to address them. The poor quality of its contemporary science, which is dominating the literature on some of the new products, is also clearly documented.^43^^,^^44^ Tobacco companies have also once again been attempting to recruit medical professionals, attend and sponsor medical conferences, and provide medical education including via Medscape.^45^ These issues are highly problematic: at a time when we urgently need to understand the impacts of emerging tobacco and nicotine products, we can’t trust the science.

### The way forward

Underpinning both the failure to recognise the conflicts between corporate interests and the public interest, and the tobacco industry’s apparent ability to reposition itself by virtue of selling products other than cigarettes, is a misconception about COIs (Box 1). An essential first step to rigorous implementation of policies to manage COIs and thus to addressing commercially driven health harms is, therefore, to engender a more accurate understanding of COIs (Box 1) – that they do not relate to a product, but to the diverging interests (and indeed remits) of industry – to maximise profits – and of public institutions – to act in the public good. The limited implementation of Article 5.3 outside ministries of health highlights that even the best COI policies will stall without this wider understanding.^27^

Applying that understanding of COIs to policymaking, it will become apparent that if a government or public institution is developing a policy that could affect sales or profitability of a product, that policy should be protected from individuals and organisations with a financial interest in that product – they are clearly conflicted. In short, regardless of the product, the affected industry should not be at the policy table, nor working in partnership with the public institution. While it is important to *develop* policy free from vested interests, it may be necessary to consult with industry at certain stages of the policy process– such as *implementation* – to enable effective regulation of its actions and products. Such interactions should be limited to those deemed ‘strictly necessary’ (Recommendation 2.1) and are ‘conducted transparently’ (Recommendation 2.2), as Article 5.3 recognises.^21^, ^27^

Similar approaches need to be applied in professional and scientific settings to prevent both individual and institutional COIs, with recent evidence indicating that universities are failing to adequately address COIs.^46^ Recent developments suggest that progress is starting to be made in both settings (see for example^47^^,^^48^), although much more needs to be done.

Another useful tool that can support public, scientific and professional organisations in identifying institutional COIs and deciding whether a collaboration with a commercial actor is appropriate is the Integrity Matrix.^11^ This involves an assessment of the practices and mission of the potential collaborator to check whether there is divergence or alignment with the mission and obligations of the public institution.

In science, as the latest developments in tobacco illustrate, we also need bolder, more structural solutions to prevent, rather than just expose, COIs. This will require new funding models that reduce the ability of harmful industries to bias science.^49^ Such models – for example, a tax on industry to create a fund that is then administered independently – already exist elsewhere.^50^ Criteria to ensure that these models can be safely administered to ensure, for example, that industry is unable to claim credit for or influence the research are essential and have also been developed.^51^ In the short term, the creation of a public database of authors’ and editors’ COIs^52^ could solve the problem of incomplete or inconsistent disclosures.

The measures outlined above are key steps towards ensuring that policy, practice and science function in the public interest and that governments, public and professional institutions can deliver on their remit. Such measures are not radical but essential to good governance. Given that the scale of commercially driven health harms is so vast that it is threatening the viability of the NHS, now is time to act.

## Funding

This article received no external financial or non-financial support. ABG is supported by ‘10.13039/100015369Local Health and Global Profits’ (Grant no MR/Y030753/1), which is part of Population Health Improvement UK (PHI UK), a national research network which works to transform health and reduce inequalities through change at the population level. The funder had no role in the design and conduct of the study; collection, management, analysis and interpretation of the data; preparation, review or approval of the manuscript; and decision to submit the manuscript for publication.

## CRediT authorship contribution statement

**Anna B Gilmore:** Writing – review & editing, Writing – original draft, Validation, Investigation, Conceptualization. **Rachel A Barry:** Writing – review & editing, Writing – original draft, Validation, Investigation. **Alice Fabbri:** Writing – review & editing, Writing – original draft, Validation, Investigation, Conceptualization.

## Declaration of competing interest

The authors declare that they have no known competing financial interests or personal relationships that could have appeared to influence the work reported in this paper.
